# Optic Disc Pit with Sectorial Retinitis Pigmentosa

**DOI:** 10.1155/2013/156023

**Published:** 2013-05-23

**Authors:** Melike Balikoglu-Yilmaz, Muhittin Taskapili, Tolga Yilmaz, Mehmet Yasin Teke

**Affiliations:** ^1^Department of Ophthalmology, Bagcilar Education and Research Hospital, 34200 Istanbul, Turkey; ^2^Ulucanlar Eye Education and Research Hospital, 34015 Ankara, Turkey

## Abstract

Sectorial retinitis pigmentosa (RP) and optic disc pit (ODP) are rare clinical conditions. We present a 40-year-old woman with a history of mild night blindness and decreased vision in the right eye for about 5 years. Fundus examination revealed retinal pigmentary changes in the superior and inferotemporal sectors covering the macula and reduced arterial calibre and ODP at the temporal edge of the optic disc. In addition, fundus autofluorescence, spectral-domain optical coherence tomography, fluorescein angiography, and multifocal electroretinogram scans confirmed these clinical findings. Visual acuity was decreased due to an atrophic-appearing foveal lesion. No intervention was suggested because of the poor visual potential. To the best of our knowledge, the present study is the first to describe coexistent optic disc pit and sectorial RP in the superior and inferotemporal sectors covering the macula in the same eye with figures.

## 1. Introduction

Optic disc pit was first defined by Wiethe in 1882 [1] and is a congenital cavitary optic disc anomaly found in approximately one in 11,000 patients [2]. It is thought to be a small localized colobomatous defect of the optic nerve head due to faulty closure of the embryonic fissure or anomalous development of the primordial optic nerve [2].

Retinitis pigmentosa (RP), also known as rod-cone dystrophy, is a heterogeneous group of inherited retinal degenerations that primarily affect rods with secondary cone degeneration and lead to tunnel vision and blindness [3]. The incidence is reported as 1 in 3000–4000 in various studies, and it is estimated to affect 1.5 to 2 million persons globally [3]. Sectorial RP was first identified by Bietti [4] and is a rare variant of RP that constitutes less than 2% of all RP subtypes [5]. 

The current report describes ODP in a patient with sectorial RP in the same eye. Associated spectral-domain optical coherence tomography (SD-OCT), fundus autofluorescence imaging, fluorescein angiography (FFA), and multifocal electroretinogram (mfERG) findings are presented. 

## 2. Case Report

A 40-year-old woman with a history of mild night blindness and decreased vision in her right eye for about 5 years was seen in our department of ophthalmology. Her medical history was not noteworthy and no history of trauma was detected. No other cases with the same symptomatology were noticed in the patient's family. At that time, visual acuity was CF 1 m and 20/20 in the right (OD) and left eyes (OS), respectively. Manifest refraction was −1.00 to −2.00 × 80° and +0.50 × 180 in OD and OS, respectively. Intraocular pressures were 16 and 15 mmHg in OD and OS, respectively. Anterior segment examination was normal in both eyes.

OD fundus examination revealed an oval, grayish crater-like depression on the temporal border of optic disc suggestive of ODP with temporal pallor, pigment epithelial atrophy, and bone spicule hyperpigmentation and pigment migration into the superior retina suggestive of superior and inferotemporal sectorial RP covering the macula. 

An atrophic-appearing foveal lesion measuring about 2 DD underlying RPE changes and narrowing of the blood vessels in the affected segment was also revealed. The unaffected regions had a normal appearance. OS fundus examination revealed no abnormalities. 

**Figure 1:**
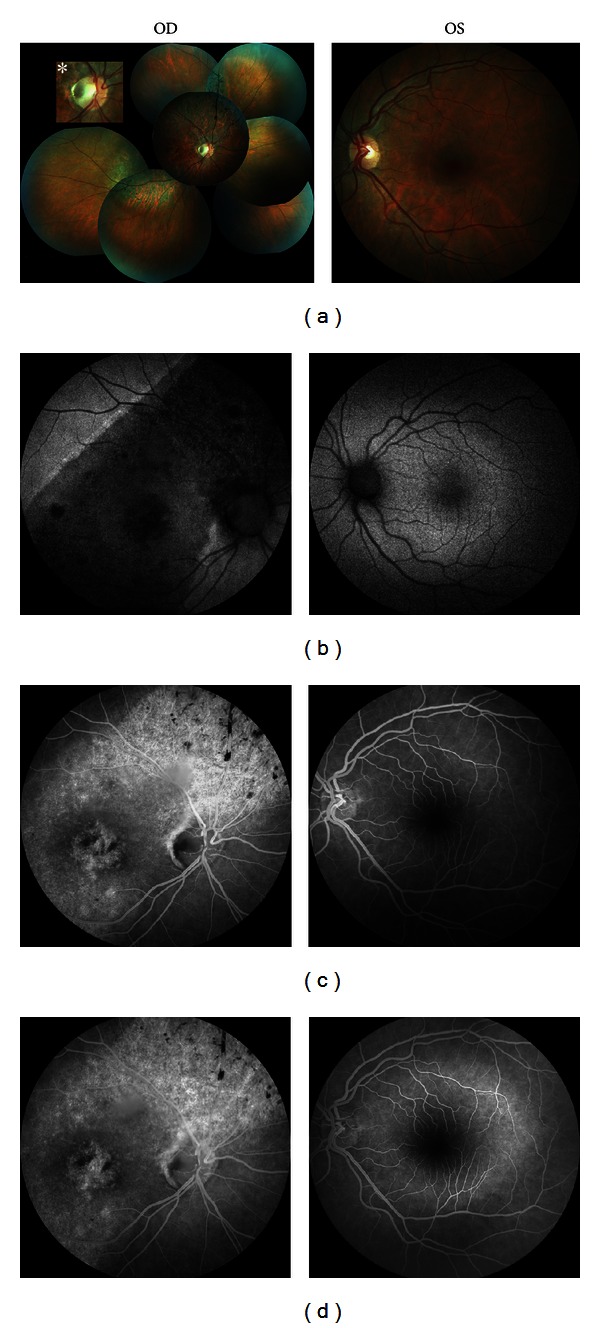
A 40-year-old woman with a history of mild night blindness and decreased vision in the right eye for about 5 years. Fundus color (a) and autofluorescence (b) photographs, and early- (c) and late-phase (d) fluorescein angiogram of this patient with unilateral optic disc pit with sectorial retinitis pigmentosa (OD indicates right eye; OS, left eye). An asterisk indicates zoomed images of the pit.

Retinal imaging with fundus color and autofluorescence photography confirmed the presence of two syndromes in OD (Figures 1(a) and 1(b)). Fundus autofluorescence (FAF) imaging showed a clear demarcation between normal and abnormal areas of the retina and hypoautoflorescence due to atrophy of the retina pigment epithelium in the region of RP, which corresponded to the area of visual field loss. 

Fluorescein angiography (FFA) (Visucam 500; Carl Zeiss Meditec AG, Jena, Germany) showed hypofluorescence at the optic pit, with adjacent retina pigment epithelium disturbance and hyperfluorescence areas due to atrophy of the retina pigment epithelium in the RP areas (Figures 1(c) and 1(d)).

**Figure 2:**
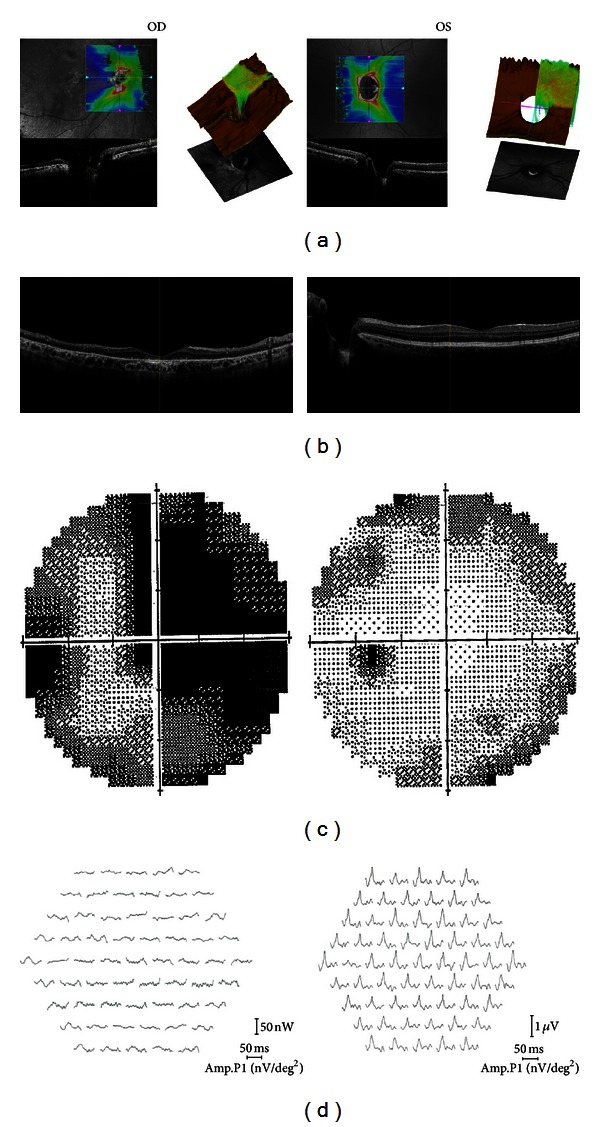
Horizontal (a), and vertical sections (b) spectral-domain optical coherence tomography images, automated Humphrey perimetry (30-2) (c), and multifocal electroretinogram (d) images of the same patient (OD indicates right eye; OS, left eye).

Advanced SD-OCT (RS-3000 Advanced OCT/SLO System; Nidek Corp., Gamagori, Japan) demonstrated a large excavation of the optic disc with a deeper temporal area in the horizontal section through the optic disc (Figure 2(a)) and thinned retina and widespread RPE atrophy in the macular and other areas in vertical cross-section through the fovea (Figure 2(b)). No posterior vitreous detachment or serous macular detachment was observed.

Automated Humphrey perimetry (30-2) revealed visual field defects compatible with the sectorial RP in OD and was normal in OS (Figure 2(c)). 

Multifocal ERG (RETIscan, Brandenburg, Germany) showed faint waves in OD and normal waveforms in OS in all rings (Figure 2(d)). 

No active intervention was suggested due to poor visual potential because of the atrophic-appearing foveal lesion. No progression of the pathologies was detected at the periodic ophthalmic examinations during the 15-month followup.

## 3. Discussion

Optic disc pit is characterized by a gray, yellow, or black oval excavation of the optic nerve head in the temporal part of the optic nerve head and is usually seen unilaterally in patients with a larger optic disc [2], as in our case. 

Optic disc pit has been associated with other abnormalities of the optic nerve, peripapillary retina, nerve fiber layer defects, or posterior vitreous detachment [2]. Complications including serous macular detachment that occurs at a frequency of 25% to 75%, macular hole, cystic changes in the macula, vision loss, and deterioration of the visual field may also be observed in ODP patients [2]. On the other hand, most patients with ODP generally have good visual acuity and are asymptomatic due to the focal nature of the RP, unless serous macular detachment occurs [2]. In our patient, there was no evidence of serous macular detachment or other complications.

Sectorial RP consists of bilateral symmetrical pigmentary changes in one or two fundus quadrants (especially the lower and nasal quadrants) [5]. Although established diagnostic criteria of RP [3] necessitate bilateral presentation, unilateral presentations and sectorial RP have also been reported [3]. The course of sectorial RP is stationary or very slowly progressive [5] while generalized RP is usually progressive. Close monitoring is, therefore, necessary to differentiate between sectorial and generalized RP. Multifocal ERG, visual field and autofluorescence documentation are also useful in the diagnosis of sectorial RP. The pigmentary changes were unilateral and involved the superior and inferotemporal quadrants covering the macula in our case. The decreased visual acuity was probably related to the atrophic-appearing foveal lesion that can occur as a consequence of sectorial RP. The upper nasal retina could not have been detached by the pit area in our patient, and retinal pigmentary changes were more prominent along the retinal veins while pigment deposits were in the “bone spicule” style, supporting sectorial RP. These changes would also have been more irregular otherwise. In addition, ancillary tests were consistent with the diagnosis of sectorial RP. 

In summary, we described an unusual case of the coexistence of ODP and sectorial RP in the same eye. Although there are some differences between the cases, the association between subtypes of RP and ODP was reported previously [6, 7]. These different ophthalmic pathologies could be present incidentally in the same eye or there might be a possible unexplained pathogenetic correlation between the two syndromes. More studies are, therefore, required. This clinical association with poor visual outcome should be kept in mind and genetic counseling should be provided in familial cases.
